# Multivariate association patterns of morpho-physiological responses in soybean under short-term drought at the early vegetative stage using a drought resistance index framework

**DOI:** 10.1186/s12870-026-09189-z

**Published:** 2026-06-13

**Authors:** Hidayat Saputra, Mochamad Arief Soleh, Jajang Sauman Hamdani, Andy Saryoko

**Affiliations:** 1 Doctorate Program of Agricultural Science, Faculty of Agriculture, Universitas Padjadjaran, Raya Bandung Sumedang St. KM 21, Jatinangor, 45363 Indonesia; 2 Department of Food Crop Production Technology, Politeknik Negeri Lampung, Soekarno-Hatta St. 10, Bandarlampung, 35144 Indonesia; 3 Department of Agronomy, Faculty of Agriculture, Universitas Padjadjaran, Raya Bandung Sumedang St. KM 21, Jatinangor, 45363 Indonesia; 4 Politeknik Enjiniring Pertanian Indonesia, Sinarmas Boulevard St. 01, Pagedangan Regency, Tangerang, Banten, 15338 Indonesia

**Keywords:** Clustering analysis, D-value, *Glycine max*, Partial least squares regression, Photosynthetic traits, Principal component analysis, Root traits

## Abstract

**Background:**

Drought stress severely limits soybean productivity, including during early vegetative growth, highlighting the importance of identifying cultivars with favorable early-stage drought responses. Because drought evaluation based on single traits may not adequately capture coordinated plant responses, integrative multivariate approaches are needed to characterize drought-response patterns among soybean cultivars.

**Methods:**

Six soybean cultivars were evaluated under greenhouse conditions following a five-day water-withholding treatment at the V3 stage. Morphological, physiological, and biochemical traits were assessed and integrated using a standardized Drought Resistance Index (DRI) and composite D-value framework. Multivariate analyses, including hierarchical clustering, correlation analysis, principal component analysis (PCA), and partial least squares regression (PLS-R), were used to evaluate coordinated drought-response patterns among cultivars.

**Results:**

Drought stress reduced most physiological and morphological traits, particularly stomatal conductance, photosynthetic performance, plant growth, and leaf water status. In contrast, proline accumulation increased under drought stress across cultivars, whereas increases in several root-related traits and transpiration responses were observed only in certain cultivars despite substantial reductions in stomatal conductance. Clear differences in drought-response profiles were observed among cultivars. Dering1 exhibited the highest D-value and, together with Dega1, was grouped within the drought-resistant response category, whereas Biosoy1 showed the lowest D-value and was classified as drought-sensitive. Correlation analysis indicated coordinated associations among plant water status, photosynthetic traits, and root-related characteristics under drought conditions. Principal component analysis revealed distinct multivariate response profiles among tolerant cultivars, with Dering1 more closely aligned with root-related and water-status-associated traits, whereas Dega1 was more strongly associated with photosynthetic and biomass-related indices. Partial least squares regression identified several traits, including net photosynthetic rate, stomatal density, instantaneous carboxylation efficiency, and root volume, as showing relatively stronger associations with D-value variation within the analytical framework.

**Conclusions:**

The integrative DRI–D-value framework, combined with multivariate analyses, provided an exploratory approach to evaluating coordinated drought-response patterns during early vegetative growth under short-term greenhouse drought conditions. The findings further suggest that early vegetative drought responses in soybean involve coordinated variation among water-status, photosynthetic, and root-related traits. Dering1 and Dega1 may therefore serve as promising candidates for further evaluation under broader developmental stages and environmental conditions.

**Supplementary Information:**

The online version contains supplementary material available at 10.1186/s12870-026-09189-z.

## Introduction

 Water scarcity caused by drought is a major constraint limiting global agricultural productivity and is becoming increasingly severe due to climate change and growing competition for water resources [1, 2]. Approximately 25% of global croplands experience substantial water limitation or irregular rainfall patterns, conditions that are expected to intensify in many agricultural regions [3, 4]. Soybean is particularly sensitive to drought stress, with reported yield losses ranging from 18.6% to 83% depending on stress severity, developmental stage, and environmental conditions [5–7]. In Southeast Asia, soybean remains an important protein source, while improving soybean performance under water-limited environments continues to be a major agricultural challenge [8–10].

Drought stress may affect soybean growth from germination through vegetative development [10, 11], particularly in tropical production systems where soybean is frequently cultivated after rice during transitional dry-to-rainy seasons [12, 13]. Under these conditions, intermittent early-season drought and declining residual soil moisture commonly expose young plants to short-term water deficit during early vegetative establishment [13, 14]. The V3 stage represents a critical period of canopy establishment, root system expansion, and vegetative growth, during which water limitation may substantially influence subsequent plant development [15, 16]. Reduced soil moisture during this stage may restrict biomass accumulation, leaf expansion, stem development, and root growth, thereby affecting plant vigor and subsequent reproductive readiness [17, 18]. Understanding morpho-physiological responses during early vegetative drought exposure is therefore important for improving early-stage drought evaluation in soybean.

In Indonesia, several soybean cultivars recognized for drought tolerance often possess relatively low yield potential and smaller seed size, limiting their broader adoption [19, 20]. Conversely, elite high-yielding cultivars generally perform well under favorable moisture conditions but frequently exhibit greater sensitivity to water deficit [21, 22]. Consequently, evaluating drought-response patterns among promising soybean cultivars under limited water availability remains important for identifying genotypes with favorable early-stage performance under drought conditions. Because drought responses involve coordinated changes across multiple physiological, morphological, and biochemical processes, integrative evaluation approaches are needed to better characterize cultivar-level response patterns [22, 23].

Morphological, physiological, and biochemical traits have frequently been associated with drought responses in soybean. Morphological traits such as root length, root volume, root-to-shoot ratio, specific leaf area, and stomatal density are commonly linked to plant water acquisition and structural adjustment under drought conditions [15, 24, 25]. Physiological traits, including relative water content, stomatal conductance, photosynthetic performance, mesophyll CO₂ diffusion, and instantaneous carboxylation efficiency, are also widely used to evaluate drought-related responses associated with plant water status and carbon assimilation [16, 26–28]. In addition, proline accumulation has often been reported as part of plant responses to osmotic stress and cellular water imbalance under drought conditions [29–31]. However, many drought evaluation studies still rely on single-trait measurements or productivity-based indices, which may not adequately capture the coordinated multivariate nature of drought-response patterns during early vegetative growth.

The Drought Resistance Index (DRI) framework provides an approach for standardizing cultivar responses under drought conditions by integrating drought resistance coefficients with trait performance under stress [32]. When combined with the composite D-value derived from principal component and membership function analyses [33, 34], the DRI–D–value framework offers a complementary multivariate approach for evaluating coordinated morpho-physiological drought-response patterns. Previous studies using drought-related indices in soybean have primarily focused on yield-based assessment, survival percentage, or single-trait evaluation approaches [35–37], whereas integrative analyses combining multiple morpho-physiological drought resistance indices during early vegetative growth remain comparatively limited. In addition, relatively few studies have examined how physiological, morphological, and root-related traits are coordinately associated within a multivariate drought-response framework under short-term water deficit conditions.

Therefore, the present study applied a standardized DRI and D-value framework to characterize drought-response patterns among promising soybean cultivars during early vegetative growth under short-term greenhouse drought conditions. The study further evaluated coordinated association patterns among morpho-physiological and biochemical drought resistance indices using hierarchical clustering, correlation analysis, principal component analysis (PCA), and partial least squares regression (PLS-R). Through this integrative approach, the study aimed to provide exploratory insight into multivariate drought-response patterns that may support future early-stage drought evaluation and cultivar validation studies in soybean.

## Materials and methods

### Plant material, experimental sites, and microclimate conditions

This study utilized six soybean cultivars. Five of these cultivars (Dering1, Dega1, Detap1, Devon1, and Anjasmoro) were acquired from the Indonesian Center for the Development and Testing of Legume Crops. The sixth cultivar, Biosoy1, was sourced from BRMP Biogen Indonesia. All cultivars featured in the experiment exhibit a determinate growth type, with an average flowering time ranging from 30 to 42 days after sowing (DAS).

The experiment was conducted from September to November 2023 in a greenhouse at the Ciparanje Experimental Farm, Faculty of Agriculture, Universitas Padjadjaran, located in Jatinangor, Sumedang Regency, West Java, Indonesia (6°54’54” S, 107°46’05” E; 700 m above sea level). The soil used as the growth medium was classified as *Inceptisol*, with the following physicochemical characteristics: soil pH 5.9, organic matter content 3.23%, total N 0.23%, C/N ratio 14, available P₂O₅ (Olsen) 6.32 ppm, available K₂O (HCl 25%) 33.51 mg/100 g, cation exchange capacity 16.22 cmol·kg⁻¹, and a loam texture (38% sand, 48% silt, 14% clay).

The mean daily air temperature inside the greenhouse during the experiment was 29.8 °C (measured using a LabJack Digit-TLH data logger). Relative humidity ranged between 36 and 49% (Digital Thermo-Hygrometer HTC-2), and light intensity ranged from 35 to 68 × 1000 lx between 08:00–09:30 a.m. and 52–79 × 1000 lx between 10:00–2:00 p.m. (measured using an Amtast-AMF023 lux meter).

### Experimental design and drought stress treatment

The experiment was arranged in a randomized complete block design (RCBD) consisting of six soybean cultivars under two water regimes (well-watered and drought-stressed conditions), with three biological replicates per treatment combination. Each replicate consisted of one pot containing a single soybean plant, which served as the experimental unit, while the three replicates also represented the experimental blocks within the RCBD. Because each pot contained only one plant, all morphological, physiological, and biochemical measurements were obtained from the individual plant representing each experimental unit. Unless otherwise specified, all data were derived from three independent biological replicates and statistical analyses were performed using replicate-level observations.

Soybean plants were cultivated in pots with a height of 25 cm and an upper diameter of 20 cm, each containing 2.8 kg of soil. The soil was collected from the topsoil layer (0–30 cm depth), air-dried, and sieved to obtain a fine, homogeneous medium free from stones and plant residues. The soil was then filled into polybags, leaving a 3 cm space from the top edge. To determine the field capacity (FC), nine pots were saturated with water, requiring approximately 900 mL per pot to reach FC. One day before sowing, all pots were watered uniformly to reach 85% of FC (equivalent to 765 mL per pot).

Four soybean seeds were sown in each pot. Six days after sowing, seedlings were thinned to retain only one healthy and uniformly growing plant per pot. All soybean plants were irrigated every two days to maintain soil moisture at 80–85% of field capacity using the weighing method until the vegetative stage V2. Crop maintenance included the application of a compound NPK fertilizer solution (50 g fertilizer dissolved in 1 L of water) at a dose of 50 mL per plant at six days after sowing, and periodic insecticide application containing the active ingredients Fipronil and Profenofos to control pest infestations.

The drought stress treatment was initiated when plants reached the V3 stage, characterized by the full expansion of the third trifoliate leaf at approximately 22 days after sowing. Drought stress was imposed by withholding irrigation for five consecutive days to simulate a short-term early vegetative water-deficit episode under greenhouse conditions. Before stress imposition, all pots assigned to the drought treatment were adjusted to a uniform initial soil moisture level of approximately 85% field capacity, as verified using a soil tester (Takemura DM-5, Takemura Electric Works Ltd., Tokyo, Japan). Soil moisture in drought-stressed pots progressively declined to approximately 78% field capacity at 1 day after stress (DAS), 62% at 3 DAS, and 49–53% field capacity at 5 DAS, corresponding to a gravimetrically determined soil water content of 21.7–23.0%. Similar short-term reductions in soil moisture during vegetative growth have been reported to induce measurable morpho-physiological responses in soybean under controlled drought conditions [38]. In the well-watered control treatment, irrigation was applied daily using the weighing method (150 mL per pot) to maintain soil moisture near field capacity throughout the experimental period.

### Measurement of morphological traits

#### Plant height and stem diameter

Plant height (cm) and stem diameter (mm) were measured before drought treatment (0 DAS) and at the end of the drought period (5 DAS) using three independent biological replicates, with each replicate represented by one plant from an individual experimental unit. The plant height was assessed by measuring from the base of the stem to the top of the shoot with a measuring tape, whereas the diameter of the stem was measured 3 cm above the base using a digital caliper. The increments in height and diameter were subsequently calculated.

#### Leaf area and specific leaf area (SLA)

Leaf area (cm²) and SLA (cm² g⁻¹) were measured at the end of the drought period (5 DAS) using three independent biological replicates, with each replicate represented by one plant from an individual experimental unit. Leaf area was determined in *ImageJ* by analyzing scanned leaf images with a calibrated scale. SLA was calculated as the ratio of total leaf area to leaf dry mass, representing the photosynthetic surface area per unit of leaf biomass, following established methods in leaf morpho-physiological studies.

#### Root length and volume

Root length (cm) and root volume (cm³) were measured at the end of the drought period (5 DAS) using three independent biological replicates, with each replicate represented by one plant from an individual experimental unit. Plants were carefully uprooted from the pots and gently washed under running water to remove adhering soil without damaging the root system. The length of the root system was determined as the distance between the stem base and the tip of the longest root, measured using a measuring tape. Root volume was determined using the water displacement method in a graduated cylinder: roots were immersed in a known volume of water, and the increase in water volume was recorded as the root volume.

#### Plant dry weight and root–shoot ratio

At the end of the drought period, plants were separated into leaves, stems, and roots, and each part was oven-dried at 85 °C until a constant weight was achieved (2–3 days). Dry weights were measured using an analytical balance with an accuracy of 0.01 g. Measurements were performed on three independent biological replicates, with each replicate represented by one plant from an individual experimental unit. The root-to-shoot ratio was calculated using the following formula:

$$\:\mathrm{R}\mathrm{o}\mathrm{o}\mathrm{t}-\mathrm{s}\mathrm{h}\mathrm{o}\mathrm{o}\mathrm{t}\:\mathrm{r}\mathrm{a}\mathrm{t}\mathrm{i}\mathrm{o}=\frac{\mathrm{r}\mathrm{o}\mathrm{o}\mathrm{t}\:\mathrm{d}\mathrm{r}\mathrm{y}\:\mathrm{w}\mathrm{e}\mathrm{i}\mathrm{g}\mathrm{h}\mathrm{t}\:\left(\mathrm{g}\right)}{\left[\mathrm{l}\mathrm{e}\mathrm{a}\mathrm{f}\:\mathrm{d}\mathrm{r}\mathrm{y}\:\mathrm{w}\mathrm{e}\mathrm{i}\mathrm{g}\mathrm{h}\mathrm{t}\:\left(\mathrm{g}\right)+\:\mathrm{s}\mathrm{t}\mathrm{e}\mathrm{m}\:\mathrm{d}\mathrm{r}\mathrm{y}\:\mathrm{w}\mathrm{e}\mathrm{i}\mathrm{g}\mathrm{h}\mathrm{t}\:\left(\mathrm{g}\right)\right]}$$

#### Stomatal density

Stomatal density was determined at the end of the drought period using three independent biological replicates, with each replicate represented by one plant from an individual experimental unit. A clear nail polish impression was made on the abaxial surface of the third fully expanded leaf from the apex, covering a 2 × 2 cm area. After drying, transparent adhesive tape was pressed onto the coated surface to lift the epidermal imprint, which was then mounted on a microscope slide. Observations were carried out between 09:00 and 11:00 a.m. using an Olympus CX33 Trinocular Microscope at 40× magnification. Stomatal density was calculated according to *Franks* [39] using the equation:

$$\:\mathrm{S}\mathrm{t}\mathrm{o}\mathrm{m}\mathrm{a}\mathrm{t}\mathrm{a}\:\mathrm{d}\mathrm{e}\mathrm{n}\mathrm{s}\mathrm{i}\mathrm{t}\mathrm{y}\:\left({\mathrm{s}\mathrm{t}\mathrm{o}\mathrm{m}\mathrm{a}\mathrm{t}\mathrm{a}\:\mathrm{m}\mathrm{m}}^{-2}\right)=\frac{\mathrm{n}\mathrm{u}\mathrm{m}\mathrm{b}\mathrm{e}\mathrm{r}\:\mathrm{o}\mathrm{f}\:\mathrm{s}\mathrm{t}\mathrm{o}\mathrm{m}\mathrm{a}\mathrm{t}\mathrm{a}}{\mathrm{m}\mathrm{i}\mathrm{c}\mathrm{r}\mathrm{o}\mathrm{s}\mathrm{c}\mathrm{o}\mathrm{p}\mathrm{i}\mathrm{c}\:\mathrm{f}\mathrm{i}\mathrm{e}\mathrm{l}\mathrm{d}\:\mathrm{a}\mathrm{r}\mathrm{e}\mathrm{a}}$$

where the field area was 0.19625 mm².

#### Measurement of physiological and biochemical traits

Physiological measurements were conducted on the third fully expanded leaf from the apex at the end of the drought period (5 DAS), using three independent biological replicates, with each replicate represented by one plant from an individual experimental unit.

#### Photosynthetic traits and gas exchange parameters

Leaf-level gas exchange measurements were performed using a portable photosynthesis system (LI-6800, LI-COR Biosciences, USA) between 09:00 and 12:00 h on the third fully expanded trifoliate leaf from the apex [40, 41] using three independent biological replicates per treatment. The measured parameters included stomatal conductance (gs; mol H₂O m⁻² s⁻¹), net photosynthetic rate (Pn; µmol CO₂ m⁻² s⁻¹), intercellular CO₂ concentration (Ci; µmol CO₂ mol⁻¹), transpiration rate (E; mmol H₂O m⁻² s⁻¹), and leaf temperature (Tleaf; °C). During measurements, chamber conditions were maintained as consistently as possible across treatments with a photosynthetic photon flux density (PPFD) of approximately 800–1000 µmol m⁻² s⁻¹, reference CO₂ concentration of 410 µmol mol⁻¹, relative humidity of approximately 60%, flow rate of 1800 µmol s⁻¹, and chamber temperature maintained near 25 °C. Water-use efficiency (WUE) was calculated as the ratio of net photosynthetic rate to transpiration rate and expressed as µmol CO₂ mmol⁻¹ H₂O. Instantaneous carboxylation efficiency (ICE) was calculated as the ratio between net photosynthetic rate (Pn) and intercellular CO₂ concentration (Ci).

#### Relative water content (RWC)

Relative water content was measured following the method of Smart and Bingham [42]. One young leaf was excised and immediately weighed to obtain its fresh weight (FW). The leaf was then immersed in distilled water and incubated in darkness for 24 h to achieve full turgidity. After blotting surface water with tissue paper, the turgid weight (TW) was recorded. The sample was then oven-dried at 85 °C for 3 days to obtain the dry weight (DW). RWC was calculated using the equation by Smart and Bingham [42]:

$$\:\mathrm{RWC}\:\left(\%\right)=\frac{\left(\mathrm{FW}-\mathrm{DW}\right)}{\left(\mathrm{TW}-\mathrm{DW}\right)}\times\:100$$

#### Leaf greenness index (SPAD) and proline content

Leaf greenness index was measured using a SPAD-502 Plus Chlorophyll Meter (Konica Minolta, Japan) on three trifoliate leaves per plant. Leaf proline accumulation was quantified using the method described by Bates [43]. Approximately 0.5 g of fresh leaf tissue was homogenized in 10 mL of 3% sulfosalicylic acid. The homogenate was reacted with 2 mL of acid ninhydrin and 2 mL of glacial acetic acid in a test tube and incubated at 100 °C for 1 h. The reaction was terminated by cooling the tubes in an ice bath. The mixture was extracted with 4 mL of toluene and vortexed for 15–20 s until phase separation occurred. The red upper toluene layer containing proline was collected using a pipette, and its absorbance was measured at 520 nm using Orion AquaMate 8000 UV–vis Spectrophotometer (Thermo Scientific, USA). Proline concentration was measured using a standard curve of pure proline and expressed on a fresh weight (FW) basis with the formula: proline (µg g⁻¹ FW)

$$\begin{aligned} \:=\frac{({\upmu\:}\mathrm{g}\:\mathrm{m}\mathrm{L}^{-1}\:\mathrm{p}\mathrm{r}\mathrm{o}\mathrm{l}\mathrm{i}\mathrm{n}\mathrm{e}\:\times\:\:\mathrm{v}\mathrm{o}\mathrm{l}\mathrm{u}\mathrm{m}\mathrm{e}\:\mathrm{o}\mathrm{f}\:\mathrm{t}\mathrm{o}\mathrm{l}\mathrm{u}\mathrm{e}\mathrm{n}\mathrm{e}\:\times\:\mathrm{v}\mathrm{o}\mathrm{l}\mathrm{u}\mathrm{m}\mathrm{e}\:\mathrm{o}\mathrm{f}\:\mathrm{s}\mathrm{u}\mathrm{l}\mathrm{f}\mathrm{o}\mathrm{s}\mathrm{a}\mathrm{l}\mathrm{i}\mathrm{c}\mathrm{y}\mathrm{l}\mathrm{i}\mathrm{c}\:\mathrm{a}\mathrm{c}\mathrm{i}\mathrm{d})}{(0.5\:\times\:115.5)} \end{aligned}$$

where 0.5 represents the sample weight (g), and 115.5 is the molecular weight of proline.

### Data and statistical analysis

#### DRI and D-value Framework

Drought resistance was evaluated using an integrated analytical framework consisting of the drought resistance coefficient (DRC), drought resistance index (DRI), membership function analysis, and the composite D-value according to Chen et al. and Du et al. [44, 45]. Briefly, trait performance under drought conditions was first standardized relative to control conditions to obtain DRC and DRI values. These standardized indices were subsequently transformed using membership functions to allow comparison among traits with different scales and biological response directions. Finally, weighted trait contributions derived from principal component analysis were integrated to calculate the composite D-value representing overall drought-response performance. Calculation of DRC, DRI values, membership functions, and composite D-values was performed in Microsoft Excel 2019.

The drought resistance coefficient (DRC) for each trait was calculated as:

$$\:\mathrm{DRC}_\mathrm{i}=\:\mathrm{DS}_\mathrm{i}/\:\mathrm{CK}_\mathrm{i}$$

where DRC_i_ represents the drought resistance coefficient of the i-th trait, DS_i_ denotes the trait value measured under drought stress conditions, and CK_i_ denotes the corresponding trait value measured under well-watered control conditions.

The drought resistance index (DRI) was calculated as follows:

$$\:\mathrm{DRI}_\mathrm{i}=\:\mathrm{DRC}_\mathrm{i}\times\left(\mathrm{DS}_\mathrm{i}/\bar{\mathrm{D}}\mathrm{S}\right)$$

where DRI_i_ represents the drought resistance index of the i-th trait, DRC_i_ represents the drought resistance coefficient, DS_i_ denotes the trait value under drought stress, and D̄S represents the mean value of the corresponding trait across all cultivars under drought conditions.

#### Membership function analysis

Trait indices were subsequently standardized using membership function analysis. Traits were categorized according to their expected biological relevance under drought conditions. Beneficial traits were those in which higher DRI values reflected improved drought-response performance, whereas cost-related traits were those in which lower values were considered favorable under water-deficit conditions. Transpiration rate (Tr), specific leaf area (SLA), and leaf temperature (LeafTemp) were treated as cost-related traits because higher transpiration rates, larger specific leaf area, and elevated leaf temperature under drought conditions may indicate lower water conservation efficiency or greater drought stress exposure during early vegetative growth.

For beneficial traits, the membership function was calculated as:

$$\:\mathrm{U}\left(\mathrm{X}_\mathrm{i}\right)=\:\left(\mathrm{X}_\mathrm{i}-{\mathrm{Xmin}}\right)/\:\left(\mathrm{Xmax}-{\mathrm{Xmin}}\right)$$

For cost-related traits, the membership function was calculated as:

$$\:\mathrm{U}\left(\mathrm{X}_\mathrm{i}\right)=\:\left(\mathrm{Xmax}-{\mathrm{X}_\mathrm{i}}\right)/\:\left(\mathrm{Xmax}-{\mathrm{Xmin}}\right)$$

where U(X_i_) represents the membership function value of the i-th trait index, X_i_ represents the observed value of the corresponding trait index, and Xmax and Xmin represent the maximum and minimum values of the corresponding trait index, respectively.

#### Weight coefficient and composite D-value

The weight coefficient for each trait index was calculated as:

$$\:\mathrm{W}_\mathrm{i}=\mathrm{P}_\mathrm{i}/\sum\mathrm{P}_\mathrm{i}$$

where W_i_ represents the weight coefficient assigned to the i-th trait index, and P_i_ represents the contribution rate of the corresponding principal component.

The composite D-value was subsequently calculated as:

$$\:\mathrm{D}=\sum\left[\mathrm{U}\left(\mathrm{X}_\mathrm{i}\right)\times\mathrm{W}_\mathrm{i}\right]$$

The D-value represents the integrated drought-response performance of each cultivar under the experimental conditions evaluated in this study.

#### Application of DRI and D-value analyses

The DRC directly describes the relative response of individual traits under drought compared with control conditions, whereas the DRI provides a standardized index emphasizing trait performance under stress relative to the overall cultivar response. In the present study, DRI values were used for correlation analysis, principal component analysis (PCA), and partial least squares regression (PLS-R). Hierarchical cluster analysis based on D-values was subsequently performed to evaluate relative drought-response groupings among soybean cultivars.

### Descriptive statistics and analysis of variance

The traits were analyzed for minimum, maximum, mean, standard deviation, and coefficient of variation (CV), and a factorial analysis of variance (ANOVA) was performed to assess the effects of drought treatment, cultivar, and their interaction. The F-test was used to determine statistical significance in the ANOVA model. In addition, Student’s t-tests were performed to compare well-watered and drought-stressed conditions within each cultivar for physiological and morphological trait responses. Statistical analyses were conducted using GraphPad Prism version 10.4.2 (GraphPad Software, California, USA, www.graphpad.com).

### Multivariate analysis approach

Correlation analysis, hierarchical clustering analysis (HCA), principal component analysis (PCA), and partial least squares regression (PLS-R) were performed using OriginPro 2026 (OriginLab Corporation, Northampton, MA, USA). The input data matrices used for each analysis differed according to the analytical objective and are specified in the respective subsections below, including the use of cultivar-level means or individual biological replicate observations where applicable.

### Correlation analysis

Pearson’s correlation analysis was conducted to explore pairwise associations among physiological, morphological, and biochemical traits, as well as their relationships with the composite D-value under both control and drought conditions. Correlation analyses were performed using replicate-level observations obtained from three biological replicates for each cultivar × treatment combination, resulting in a total of 18 observations (6 cultivars × 3 biological replicates). This analysis aimed to identify shifts in the direction and strength of trait associations induced by water deficit and to assess coordinated multi-trait response patterns under drought conditions. In addition, correlation analysis among drought resistance index (DRI) traits and the composite D-value was performed to evaluate relative association patterns within the integrated drought-response framework. Because the D-value was mathematically derived from DRI trait values, these associations were interpreted cautiously as exploratory assessments of relative association structure rather than as evidence of independent causal relationships.

### Principal component analysis (PCA) and cluster analysis

Principal component analysis (PCA) was conducted using cultivar-level mean drought resistance index (DRI) values to summarize multivariate variation and evaluate overall drought-response association patterns among soybean cultivars under the evaluated experimental conditions. Hierarchical clustering analysis (HCA) was performed using replicate-level D-values obtained from three biological replicates for each cultivar, resulting in a total of 18 observations (6 cultivars × 3 biological replicates). Ward’s minimum variance method with Euclidean distance as the dissimilarity metric was applied to evaluate relative grouping patterns among cultivars based on D-value similarity.

### Partial least squares regression (PLS-R)

Partial least squares regression (PLS-R) was performed using drought resistance index (DRI) values from individual biological replicates across all cultivars under drought conditions (6 cultivars × 3 biological replicates; *n* = 18 observations). The analysis was intended to examine covariance relationships between DRI traits and the composite D-value within the integrated drought-response framework. Predictor variables were standardized prior to analysis to minimize scale differences among traits. Model validation was conducted using leave-one-out cross-validation (LOOCV), in which one observation was iteratively excluded from model calibration and subsequently predicted using the remaining observations. A two-component PLS-R model was selected based on model parsimony and LOOCV-derived Q² performance, as additional latent variables provided only marginal improvement relative to increased model complexity. Because the number of observations was relatively limited compared with the number of predictor variables and the D-value was derived from the evaluated DRI traits, the PLS-R analysis was interpreted as an exploratory multivariate approach. Variable Importance in Projection (VIP) values were subsequently used to identify traits showing relatively stronger associations with D-value variation within the analytical framework.

## Results

### Effects of cultivar, water condition, and their interaction on soybean traits

Analysis of variance revealed that soybean responses to well-watered and drought-stress conditions differed across the evaluated morpho-physiological traits (Table 1). Drought treatment exerted highly significant effects (*p* < 0.01) on most measured traits. The cultivar factor significantly influenced several physiological traits, including Pn (*p* < 0.05), RWC, and proline (*p* < 0.01), as well as morphological traits such as Height, RootVol, LeafArea, and RootDW (*p* < 0.01). Significant cultivar × drought interactions were detected for RWC, Height, and RootDW (*p* < 0.01), as well as RootVol (*p* < 0.05), indicating differential cultivar responses under water-deficit conditions. Overall, drought stress reduced several physiological and morphological traits during the early vegetative stage. These results provided the statistical basis for subsequent multivariate analyses examining variation in cultivar-level drought-response patterns under the evaluated experimental conditions.

**Table 1:** Analysis of variance for different soybean cultivars under drought stress

Trait	Cultivar (C)	Drought stress (DS)	C x DS
gs	0.742^ns^	568.890^**^	0.757^ns^
Pn	2.666^*^	92.414^**^	0.819^ns^
Tr	0.539^ns^	0.091^ns^	0.687^ns^
WUE	0.847^ns^	21.917^**^	1.370^ns^
Ci	0.739^ns^	25.140^**^	0.454^ns^
ICE	2.483^ns^	86.955^**^	0.781^ns^
SPAD	1.676^ns^	35.450^**^	1.508^ns^
RWC	25.819^**^	320.776^**^	20.243^**^
LeafTemp	0.645ns	132.220^**^	0.891^ns^
Proline	8.287^**^	10.203^**^	0.477^ns^
Height	23.392^**^	1538.383^**^	65.556^**^
StemDia	1.657^ns^	41.007^**^	0.742^ns^
RootLen	2.487^ns^	4.475^*^	0.692^ns^
RootVol	4.602^**^	6.471^*^	3.836^*^
LeafArea	4.292^**^	21.784^**^	1.930^ns^
SLA	2.157^ns^	0.236^ns^	0.584^ns^
StomaDen	6.116^**^	14.473^**^	0.199^ns^
ShootDW	8.395^**^	17.959^**^	0.421^ns^
RootDW	8.542^**^	2.894^**^	4.381^**^
Biomass	11.203^**^	20.929^**^	1.022^ns^
RootShoot	2.202^ns^	3.641^ns^	1.760^ns^

*gs *Stomatal conductance, *Pn *Net photosynthetic rate, *Tr *Transpiration rate, *WUE *Water use efficiency, *Ci *Intercellular CO₂ concentration, *ICE *Instantaneous carboxylation efficiency, *SPAD *Leaf greenness index, *RWC *Relative water content, *LeafTemp *Leaf surface temperature, *Proline *Leaf proline content, *Height *Plant height increment, *StemDia *Stem diameter increment, *RootLen *Root length, *RootVol *Root volume, *LeafArea *Total leaf area plant^− 1^, *SLA *Specific leaf area, *StomaDen *Stomatal density, *ShootDW *Shoot dry weight, *RootDW *Root dry weight, *Biomass *Dry weight plant^− 1^, *RootShoot *Root-to-shoot ratio. ns: non-significant at α = 0.05; *: *p* < 0.05; **: *p* < 0.01

### Descriptive changes in morpho-physiological traits under drought stress

Drought stress reduced the mean values of most soybean morpho-physiological traits, particularly net photosynthetic rate (Pn), stomatal conductance (gs), relative water content (RWC), plant height (Height), leaf area, leaf greenness index (SPAD), intercellular CO₂ concentration (Ci), and biomass-related traits (Table 2). Declines in Pn, RWC, Height, StemDia, RootDW, RootShoot, and ICE were accompanied by increased coefficients of variation (CV), indicating greater variation among cultivars under drought conditions. In contrast, reductions in gs, leaf area, and SPAD occurred with relatively stable CV values, suggesting more uniform response patterns among cultivars. Leaf temperature and proline content generally increased under drought stress, whereas RootShoot responses varied among cultivars. Overall, the observed variation in trait responses under drought conditions highlights the potential usefulness of these morpho-physiological characteristics for describing early vegetative drought-response patterns among soybean cultivars under the evaluated greenhouse conditions.

**Table 2:** Descriptive statistical analysis of soybean morpho-physiological traits

Treatment	Trait	Min	Max	Mean	SD	CV%
Control	gs	0.92	1.86	1.53	0.23	15.20
	Pn	6.22	15.40	10.85	2.37	21.89
	Tr	1.32	6.57	3.44	1.76	51.22
	WUE	1.71	8.26	4.06	2.19	53.86
	Ci	340.68	382.26	361.22	13.02	3.61
	ICE	0.02	0.04	0.03	0.01	19.87
	SPAD	32.00	45.50	40.30	2.88	7.14
	RWC	80.00	84.50	82.26	1.30	1.57
	LeafTemp	28.40	34.90	31.39	1.89	6.01
	Proline	2.91	17.04	8.79	3.45	39.29
	Height	9.30	14.90	12.38	1.53	12.37
	StemDia	0.18	0.81	0.41	0.17	42.73
	RootLen	20.00	44.50	30.71	6.60	21.49
	RootVol	1.00	2.50	1.79	0.41	22.90
	LeafArea	218.18	376.20	284.24	39.99	14.07
	SLA	265.53	613.62	446.55	97.85	21.91
	StomaDen	142.68	254.78	199.58	33.63	16.85
	ShootDW	0.71	1.89	1.15	0.30	25.69
	RootDW	0.11	0.29	0.21	0.05	26.06
	Biomass	0.83	2.14	1.36	0.33	24.29
	RootShoot	0.12	0.25	0.18	0.04	22.84
Drought	gs	0.12	0.20	0.16	0.02	14.12
	Pn	2.18	7.91	4.81	1.74	36.21
	Tr	1.41	7.91	3.63	1.68	46.34
	WUE	0.45	4.04	1.52	0.82	54.12
	Ci	296.37	364.71	336.27	14.94	4.44
	ICE	0.01	0.02	0.01	0.01	35.19
	SPAD	29.90	39.80	35.11	2.79	7.94
	RWC	53.85	80.71	69.40	8.23	11.86
	LeafTemp	35.10	39.20	37.54	1.11	2.97
	Proline	6.24	20.24	11.77	4.41	37.47
	Height	3.50	9.20	6.49	1.80	27.71
	StemDia	0.08	0.24	0.14	0.04	31.38
	RootLen	18.60	37.80	26.69	5.71	21.39
	RootVol	0.80	2.10	1.54	0.43	27.94
	LeafArea	163.34	298.10	238.33	34.98	14.68
	SLA	256.71	631.23	461.49	96.61	20.93
	StomaDen	96.82	244.59	163.34	39.22	24.01
	ShootDW	0.49	1.28	0.90	0.21	23.83
	RootDW	0.11	0.33	0.19	0.06	33.10
	Biomass	0.60	1.50	1.08	0.24	22.09
	RootShoot	0.11	0.36	0.22	0.07	32.35

*gs *Stomatal conductance, *Pn *Net photosynthetic rate, *Tr *Transpiration rate, *WUE *Water use efficiency, *Ci *Intercellular CO₂ concentration, *ICE *Instantaneous carboxylation efficiency, *SPAD *Leaf greenness index, *RWC *Relative water content, *LeafTemp *Leaf surface temperature, *Proline *Leaf proline content, *Height *Plant height increment, *StemDia *Stem diameter increment, *RootLen *Root length, *RootVol *Root volume, *LeafArea *Total leaf area plant^− 1,^*SLA *Specific leaf area, *StomaDen *Stomatal density, *ShootDW *Shoot dry weight, *RootDW *Root dry weight, *Biomass *Dry weight plant^− 1^, *RootShoot *Root-to-shoot ratio

### Variation responses of cultivars in physiological traits under control and drought stress

**Fig. 1:**
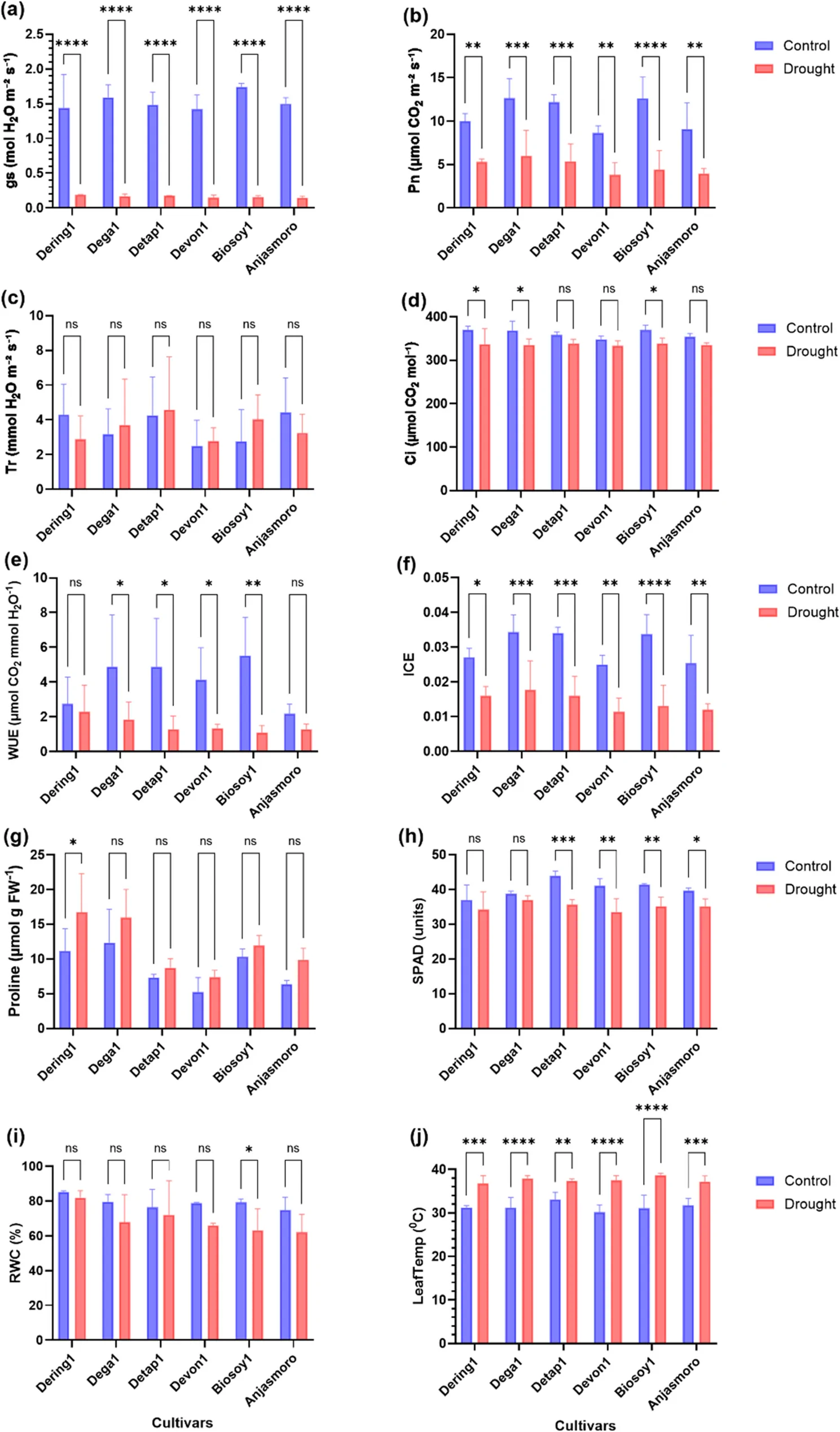
Responses of (**a**) gs: stomatal conductance, (**b**) Pn: net photosynthesis, (**c**) Tr: transpiration rate, (**d**) Ci: intercellular CO_2_, (**e**) WUE: water use efficiency, (**f**) ICE: instantaneous carboxylation efficiency, (**g**) proline: leaf proline content, (**h**) SPAD: leaf greenness index, (**i**) RWC: leaf relative water content, and (**j**) LeafTemp: leaf surface temperature in soybean plant cultivars (*Glycine max* L. Merr) exposed to control (well-watered) and drought stress. Bars represent mean value ± SE (*n* = 3). ns: non-significant at α = 0.05, *p* < 0.05 (*), *p* < 0.01 (**), *p* < 0.001 (***), and *p* < 0.0001 (****) are significant differences between control and drought treatments within the same cultivar based on Student’s t-test and no statistical comparisons among cultivars within the same water conditions

Drought stress generally reduced net photosynthetic rate (Pn) and stomatal conductance (gs) across cultivars relative to control conditions. gs declined from 1.42 to 1.74 mol H₂O m⁻² s⁻¹ under control conditions to 0.15–0.18 mol H₂O m⁻² s⁻¹ under drought (Fig. 1a). Relative reductions in gs were 87.3% in Dering1, 89.2% in Dega1, 88.2% in Detap1, 89.5% in Devon1, 90.8% in Biosoy1, and 90.1% in Anjasmoro. Similarly, Pn decreased from 8.64 to 12.63 µmol CO₂ m⁻² s⁻¹ under control conditions to 3.81–5.98 µmol CO₂ m⁻² s⁻¹ under drought (Fig. 1b). Relative reductions in Pn were 47.0% in Dering1, 52.7% in Dega1, 55.8% in Detap1, 55.9% in Devon1, 64.9% in Biosoy1, and 56.2% in Anjasmoro.

Transpiration rate (Tr) showed contrasting response patterns among cultivars under drought stress (Fig. 1c). In some cultivars, Tr increased relative to control conditions, rising from 2.50 to 4.25 mmol H₂O m⁻² s⁻¹ under control conditions to 2.79–4.58 mmol H₂O m⁻² s⁻¹ under drought. Relative increases in Tr were 17.1% in Dega1, 7.8% in Detap1, 11.3% in Devon1, and 46.1% in Biosoy1. In contrast, Tr decreased in Dering1 and Anjasmoro under drought, with relative reductions of 36.2% and 26.6%, respectively, compared with their corresponding control conditions. The increase in Tr observed in several cultivars under drought occurred concurrently with substantial reductions in gs. This pattern was accompanied by relatively higher leaf vapor pressure deficit (VPD_leaf) values and greater leaf-to-air temperature differences (ΔTleaf–Tair) under drought conditions, particularly in cultivars exhibiting increased Tr responses despite reduced gs. In these cultivars, ΔTleaf–Tair values ranged from approximately 2.9 to 4.0 °C and VPD_leaf reached 1.5–1.8 kPa, whereas cultivars showing reduced Tr generally exhibited lower ΔTleaf–Tair values (approximately 1.0–2.3 °C) and lower VPD_leaf values (approximately 1.2–1.3 kPa), compared with average control values of approximately 1.1 kPa (Fig.S1-S2).

Water-use efficiency (WUE), intercellular CO₂ concentration (Ci), and instantaneous carboxylation efficiency (ICE) generally declined under drought conditions, although the magnitude of reduction varied among cultivars. Ci decreased by 4.2–9.0% relative to control conditions, from 347.91 to 369.84 to 333.43–338.85 µmol CO₂ mol⁻¹ (Fig. 1d). WUE decreased from 2.19 to 5.52 µmol CO₂ mmol⁻¹ H₂O under control conditions to 1.09–2.29 µmol CO₂ mmol⁻¹ H₂O under drought (Fig. 1e). Relative reductions in WUE were 16.4% in Dering1, 62.3% in Dega1, 73.8% in Detap1, 67.6% in Devon1, 80.3% in Biosoy1, and 41.1% in Anjasmoro. ICE declined from 0.025 to 0.034 under control conditions to 0.011–0.018 under drought (Fig. 1f). Relative reductions in ICE were 41.1% in Dering1, 48.5% in Dega1, 53.5% in Detap1, 54.3% in Devon1, 62.0% in Biosoy1, and 53.6% in Anjasmoro.

Leaf proline content increased in all cultivars under drought stress (Fig. 1g). Under control conditions, proline content ranged from 5.27 to 12.35 µmol g⁻¹ fresh weight and increased to 7.39–15.95 µmol g⁻¹ fresh weight under drought conditions. Relative increases in proline content were 50.1% in Dering1, 29.1% in Dega1, 40.3% in Detap1, 15.8% in Biosoy1, and 56.3% in Anjasmoro. Leaf greenness index (SPAD) generally decreased under drought conditions (Fig. 1h). Relative reductions in SPAD values were 7.2% in Dering1, 4.6% in Dega1, 18.8% in Detap1, 18.5% in Devon1, 15.0% in Biosoy1, and 11.6% in Anjasmoro.

RWC declined from 80.6 to 83.8% under control conditions to 56.4–79.6% under drought (Fig. 1i). Drought stress significantly reduced leaf relative water content (RWC), with reductions were 5.0% in Dering1, 9.1% in Dega1, 10.4% in Detap1, 18.2% in Devon1, 31.7% in Biosoy1, and 19.6% in Anjasmoro. Leaf temperature increased under drought across cultivars compared with control conditions (average 32.7 °C) (Fig. 1j), reaching 36.7 °C in Dering1, 37.9 °C in Dega1, 37.4 °C in Detap1, 37.5 °C in Devon1, 38.6 °C in Biosoy1, and 37.2 °C in Anjasmoro under drought stress.

### Variation responses of cultivars in morphological traits under normal and drought stress

**Fig. 2:**
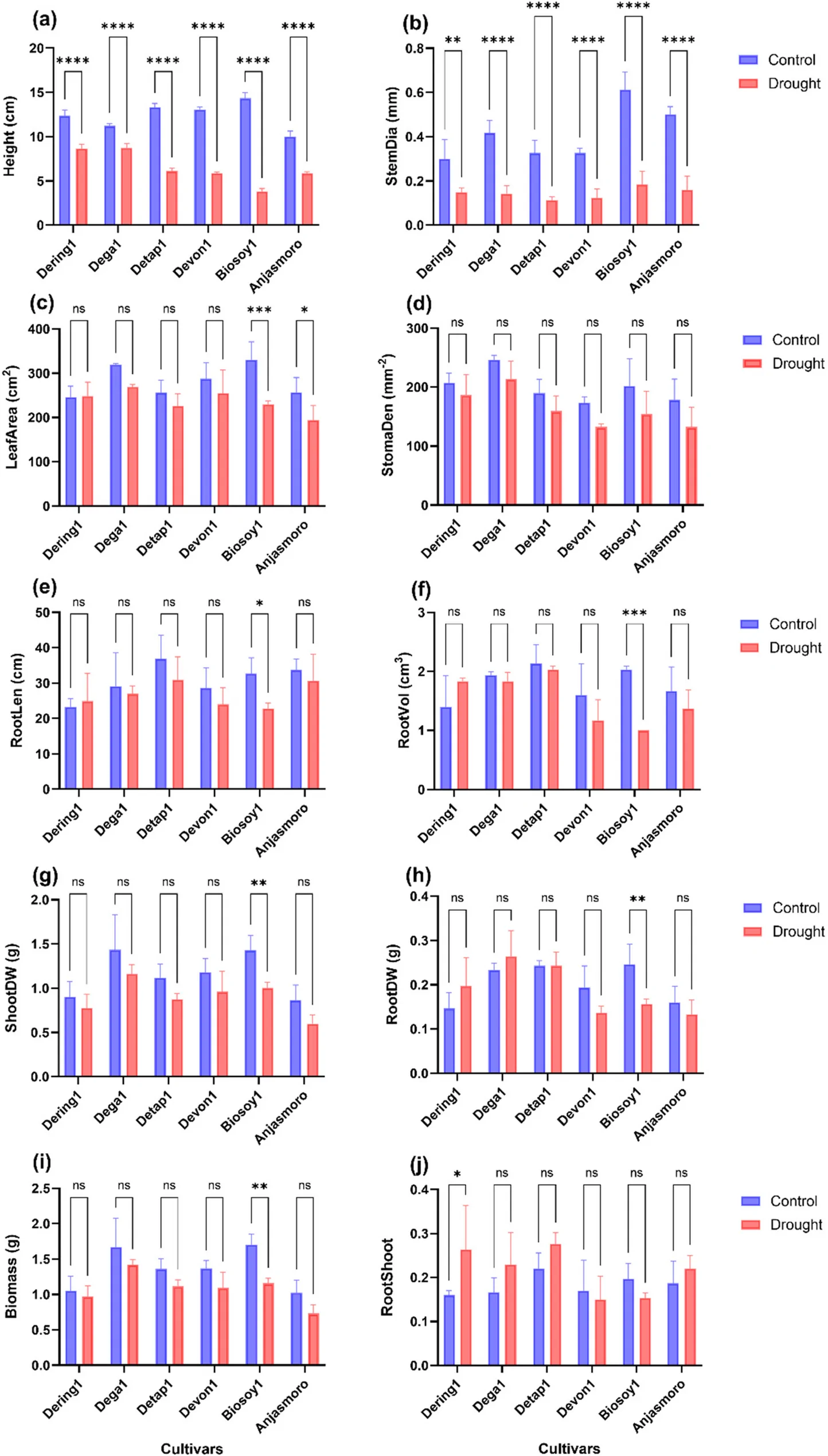
Responses of (**a**) height increment, (**b**) stem diameter increment, (**c**) leaf area plant^− 1^, (**d**) stomatal density, (**e**) root length, (**f**) root volume, (**g**) shoot dry weight, (**h**) root dry weight, (**i**) biomass plant^− 1^, and (**j**) root to shoot ratio in soybean plant cultivars (*Glycine max* L. Merr) exposed to control (well-watered) and drought stress. Bars represent mean value ± SE (*n* = 3). ns: non-significant at α = 0.05, *p* < 0.05 (*), *p* < 0.01 (**), *p* < 0.001 (***), and *p* < 0.0001 (****) are significant differences between control and drought treatments within the same cultivar based on Student’s t-test and no statistical comparisons among cultivars within the same water conditions

Under control conditions, soybean plants exhibited height increments ranging from 10.0 to 14.3 cm, which declined to 3.8–8.7 cm under drought stress (Fig. 2a). Relative reductions in plant height ranged from 22.3% in Dega1 to 74.6% in Biosoy1 compared with their corresponding control conditions. Stem diameter (StemDia) also declined under drought, decreasing from 0.33 to 0.61 mm under control conditions to 0.11–0.18 mm under drought (Fig. 2b). Relative reductions in StemDia ranged from 50.0% in Dering1 to 70.1% in Biosoy1.

Leaf area decreased from 245.6 to 330.5 cm² under control conditions to 194.6–268.6 cm² plant⁻¹ under drought stress (Fig. 2c). Relative reductions ranged from 11.4% in Devon1 to 30.6% in Biosoy1. Stomatal density also decreased across cultivars from 173.2 to 246.3 stomata mm⁻² under control conditions to 132.5–214.0 stomata mm⁻² under drought (Fig. 2d), corresponding to relative reductions ranging from 9.8% in Dering1 to 25.7% in Anjasmoro.

Root length (RootLen) decreased in most cultivars under drought stress, from 23.3 to 36.8 cm under control conditions to 22.8–30.9 cm under drought (Fig. 2e). Relative reductions ranged from 7.4% in Dega1 to 30.3% in Biosoy1 compared with their respective control conditions. In contrast, Dering1 exhibited a 6.9% increase in RootLen under drought relative to its control condition.

Root volume (RootVol) generally declined under drought stress, decreasing from 1.40 to 2.13 cm³ under control conditions to 1.00–2.03 cm³ under drought (Fig. 2f). Relative reductions in RootVol ranged from 4.7% in Detap1 to 50.0% in Biosoy1. In contrast, Dering1 showed an increase of approximately 31% in RootVol under drought relative to its control condition.

Shoot dry weight (ShootDW) declined under drought stress from 0.90 to 1.43 g under control conditions to 0.67–1.16 g under drought (Fig. 2g), corresponding to relative reductions ranging from 14.0% in Dering1 to 29.7% in Biosoy1. In contrast, root dry weight (RootDW) responses varied among cultivars under drought conditions (Fig. 2h). Relative increases in RootDW were observed in Dering1 (34.1%) and Dega1 (12.9%) compared with their corresponding control conditions, whereas RootDW remained relatively unchanged in Detap1. Relative reductions in RootDW were observed in Anjasmoro (14.5%), Devon1 (29.3%), and Biosoy1 (43.4%).

Total plant dry weight (biomass) decreased from 1.05 to 1.70 g plant⁻¹ under control conditions to 0.83–1.42 g plant⁻¹ under drought stress (Fig. 2i), corresponding to relative reductions ranging from 7.3% in Dering1 to 31.9% in Biosoy1. Changes in ShootDW and RootDW were accompanied by variation in root-to-shoot ratio (RootShoot) responses among cultivars (Fig. 2j). Relative increases in RootShoot were observed in Anjasmoro (15.0%), Detap1 (25.8%), Dega1 (38.0%), and Dering1 (64.6%) compared with their respective control conditions. In contrast, RootShoot decreased by 11.8% in Devon1 and 22.0% in Biosoy1 under drought stress.

#### Evaluation of cultivar-level drought-response patterns using the D-value framework

**Fig. 3:**
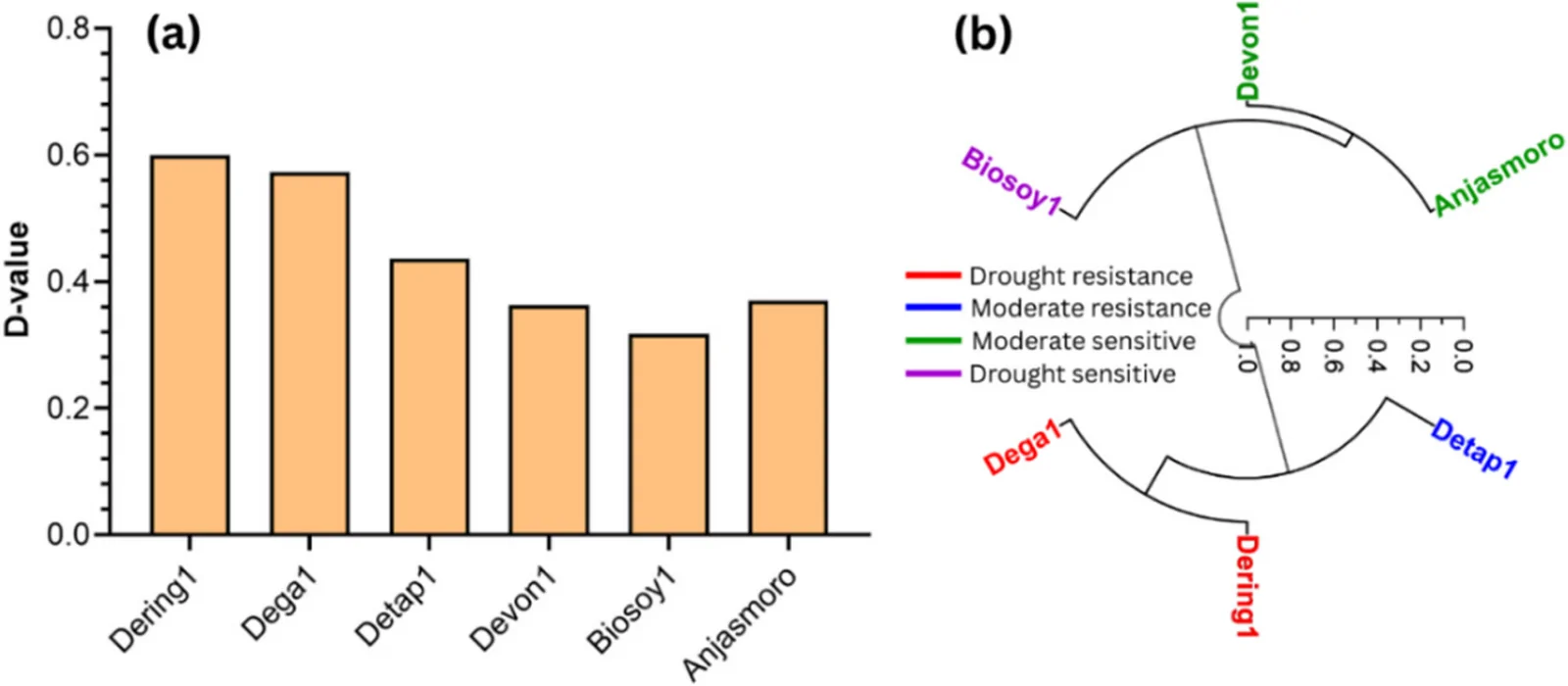
**(a)** D-value ranking and **(b)** hierarchical clustering analysis of D-values among soybean cultivars under short-term drought conditions at the early vegetative stage. Cluster categories shown in panel (**b**) represent relative drought-response groupings derived from the D-value framework under the evaluated greenhouse conditions

Using the integrated D-value framework derived from drought resistance index (DRI) and membership function analyses, clear differences in D-value profiles and cultivar-level drought-response patterns were observed among soybean cultivars (Fig. 3a). The D-values ranged from 0.32 to 0.60. Dering1 exhibited the highest D-value (0.60), followed by Dega1 (0.57), whereas Biosoy1 showed the lowest D-value (0.32). Detap1 ranked third (0.44), while Anjasmoro (0.37) and Devon1 (0.36) occupied intermediate positions.

Hierarchical clustering based on Euclidean distances derived from replicate-level D-values separated the six soybean cultivars into four relative drought-response categories under short-term drought conditions (Fig. 3b): drought-resistant, moderately drought-resistant, moderately drought-sensitive, and drought-sensitive response groups. Dering1 and Dega1 were classified within the drought-resistant response group and exhibited the highest D-values (0.57–0.60). Detap1 was positioned within the moderately drought-resistant response group with a D-value of 0.44. Anjasmoro and Devon1 formed the moderately drought-sensitive response group, with D-values ranging from 0.36 to 0.37, whereas Biosoy1 was classified within the drought-sensitive response group and exhibited the lowest D-value (0.32) under the evaluated greenhouse conditions.

### Correlation analysis of morpho-physiological traits among different soybean cultivars at control and drought conditions

**Fig. 4:**
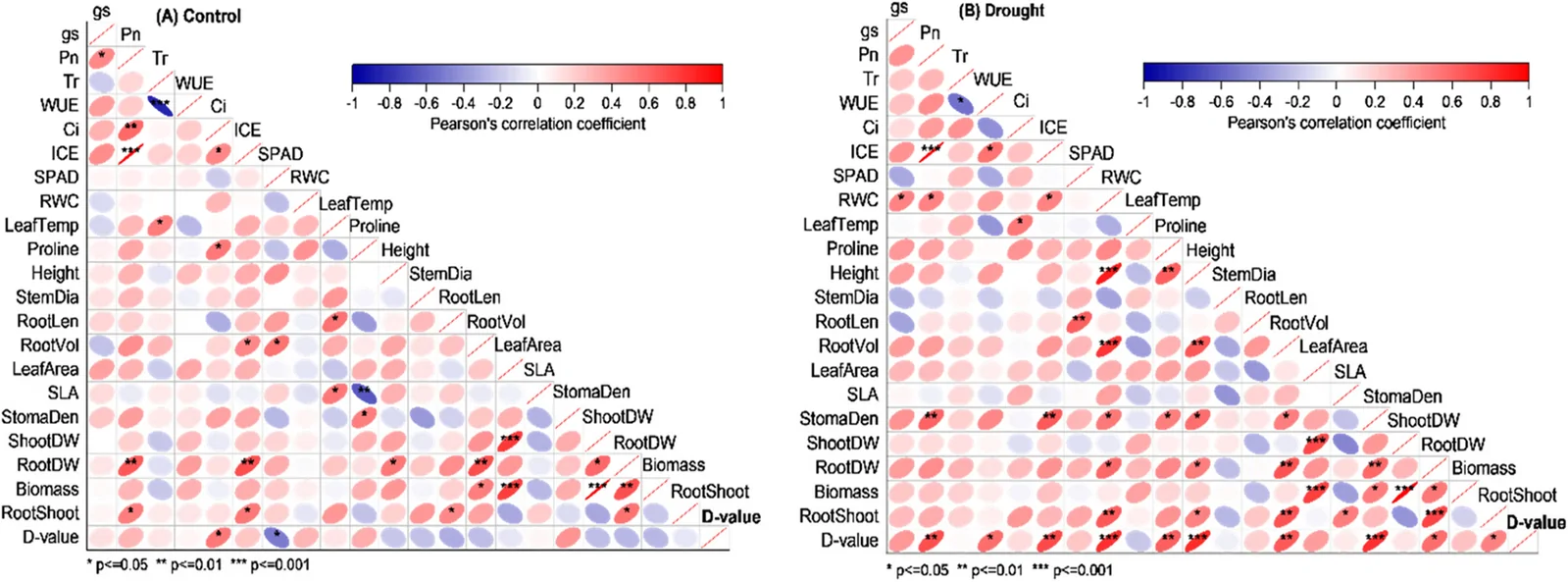
Heatmap of the correlation matrix among physiological and morphological traits of soybean and their relationships with the D-value under normal water (control) conditions (**A**) and drought stress (**B**). Red indicates positive correlations, whereas blue indicates negative correlations. Color intensity and ellipse shape reflect correlation strength; darker colors and more elongated ellipses represent stronger correlations, while ellipses approaching a circular shape indicate weak or near-zero correlations

Correlation analysis was performed to examine the relationships among 21 morpho-physiological traits and the composite D-value under two water regimes: well-watered (control) and drought stress (Fig. 4). The association patterns between the D-value and measured traits differed between the two conditions. Under control conditions, the D-value showed significant positive correlations with Ci (*p* < 0.01) and positive associations with Pn, RWC, proline, and stomatal density (StomaDen). In contrast, several root-related traits, biomass components, and SPAD tended to show weaker or negative associations with the D-value under well-watered conditions (Fig. 4A).

Under drought stress, the correlation pattern shifted substantially. The D-value showed significant positive correlations (*p* < 0.05) with WUE, RootDW, and RootShoot, and highly significant positive correlations (*p* < 0.01 to *p* < 0.001) with RootVol, RWC, StomaDen, Pn, ICE, proline, and Height (Fig. 4B). In contrast, leaf temperature (LeafTemp) was negatively correlated with the D-value. Compared with the control condition, drought stress was associated with stronger positive relationships between the D-value and several root, physiological, and biochemical traits. These patterns indicate shifts in trait association structure under water-deficit conditions, with greater alignment of several root-related and physiological traits with D-value variation during drought stress.

#### Correlation analysis of drought resistance indices associated with morpho-physiological traits across soybean cultivars

**Fig. 5:**
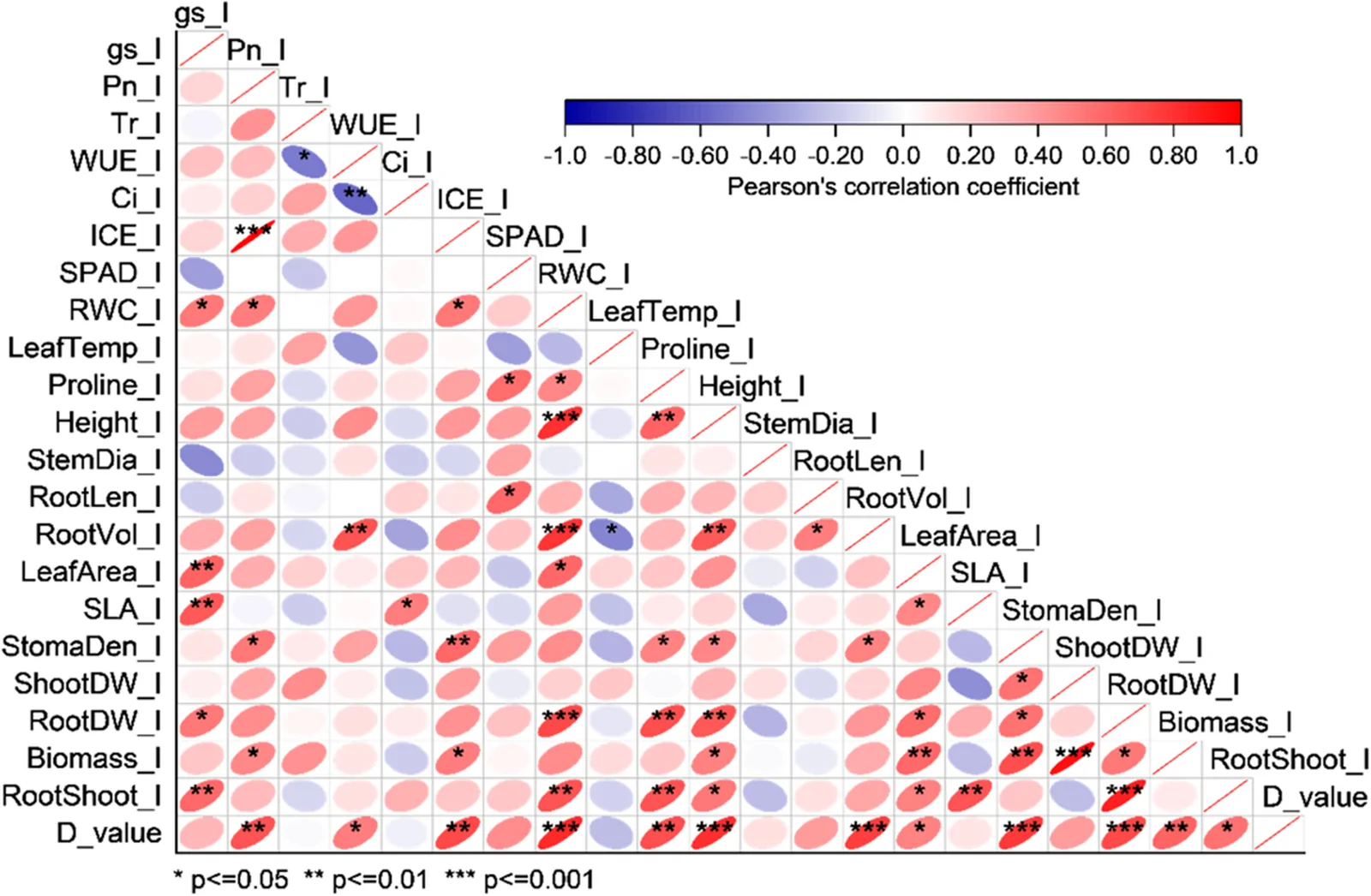
Heatmap of correlations among drought resistance indices (DRI), morpho-physiological traits, and the D-value. Red indicates positive correlations, whereas blue indicates negative correlations. Color intensity and ellipse shape represent the strength of the correlations; darker colors and more elongated ellipses denote stronger correlations, while ellipses approaching a circular shape indicate weak or near-zero correlations

The correlation analysis revealed multiple significant to highly significant positive associations among drought resistance indices (DRI) of the evaluated traits (Fig. 5). The relative water content index (RWC_I) showed significant positive correlations with gs_I, Pn_I, ICE_I, Proline_I, RootVol_I, LeafArea_I, Height_I, RootDW_I, and RootShoot_I. Proline_I was positively correlated (*p* < 0.01) with SPAD_I and StomaDen_I, and showed highly significant positive associations (*p* < 0.001) with RootDW_I, Height_I, and RootShoot_I. The stomatal conductance index (gs_I) was positively associated (*p* < 0.01) with RWC_I, LeafArea_I, SLA_I, RootDW_I, and RootShoot_I. Similarly, Pn_I showed positive correlations (*p* < 0.01) with ICE_I, RWC_I, StomaDen_I, and Biomass_I. StomaDen_I also exhibited significant positive associations (*p* < 0.01) with ICE_I, Height_I, RootVol_I, ShootDW_I, RootDW_I, and Biomass_I.

Several root-related indices exhibited strong association patterns with other DRI traits. RootVol_I showed positive correlations (*p* < 0.01) with RootLen_I and StomaDen_I, and highly significant positive correlations (*p* < 0.001) with WUE_I and RWC_I. In contrast, RootVol_I was negatively correlated (*p* < 0.01) with LeafTemp_I, while LeafTemp_I showed a positive association with Tr_I. RootDW_I exhibited significant to highly significant positive associations (*p* < 0.01 to *p* < 0.001) with Height_I, LeafArea_I, Biomass_I, and RootShoot_I. Significant negative associations (*p* < 0.01) were observed between WUE_I and Tr_I, as well as between RootVol_I and LeafTemp_I, whereas a highly significant negative correlation (*p* < 0.001) was detected between WUE_I and Ci_I.

With respect to the composite drought resistance score (D-value), WUE_I and LeafArea_I showed significant positive correlations (*p* < 0.01), whereas Pn_I, ICE_I, RWC_I, Proline_I, RootVol_I, StomaDen_I, Height_I, RootDW_I, and Biomass_I exhibited highly significant positive correlations (*p* < 0.001). Overall, the association patterns between DRI traits and the D-value were generally consistent with those observed between raw trait values and the D-value under drought conditions. Because the D-value was mathematically derived from integrated DRI trait values, these correlations were interpreted as exploratory association patterns within the analytical framework rather than as evidence of independent causal relationships. Collectively, the results indicate coordinated association patterns among multiple morpho-physiological and root-related drought resistance indices during the early vegetative stage of soybean under drought conditions.

## Principal component analysis of drought resistance indices in different soybean cultivars

**Fig. 6:**
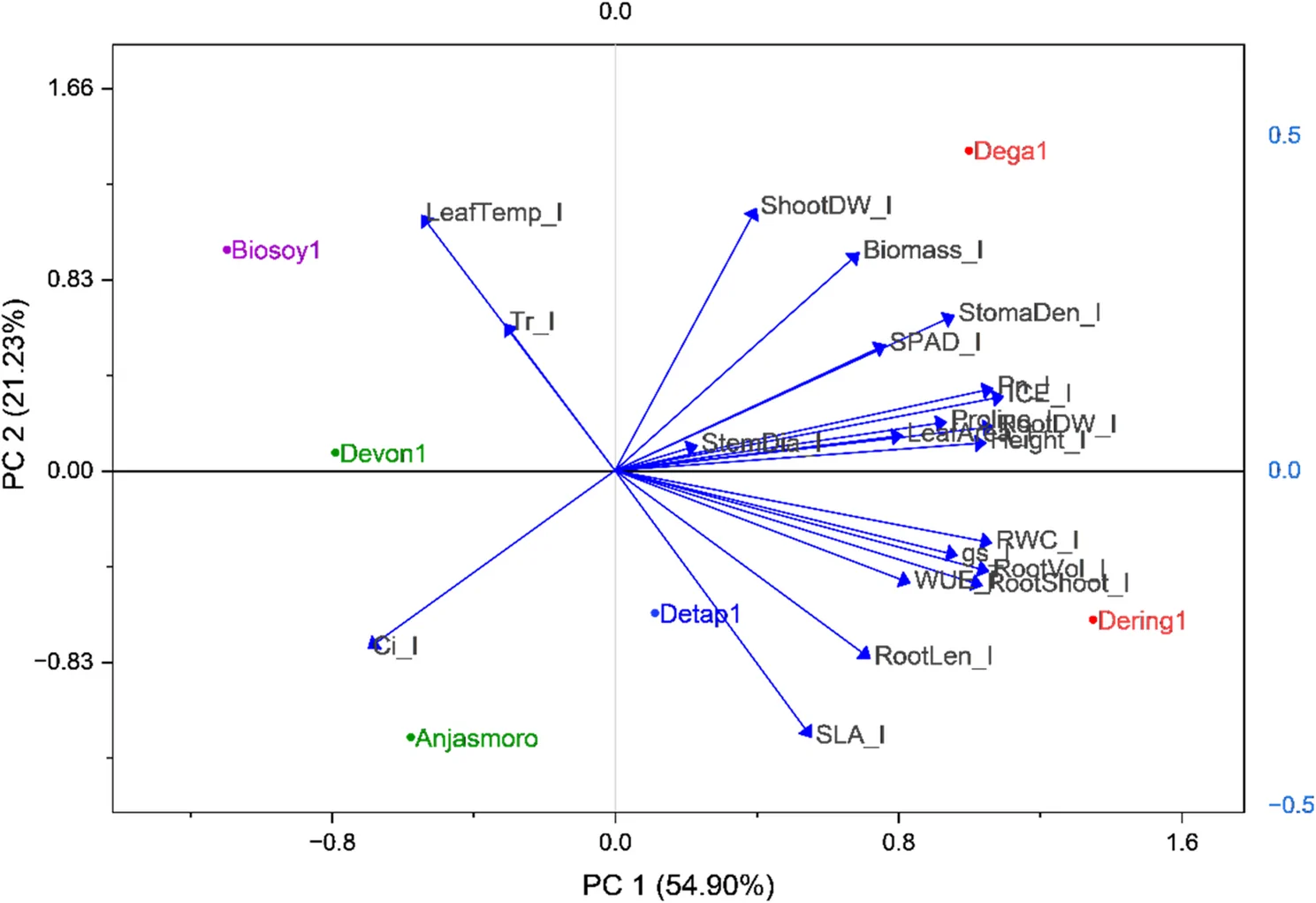
Principal component analysis (PCA) biplot of PC1 and PC2 derived from 21 drought resistance index (DRI) traits across six soybean cultivars under short-term drought conditions at the early vegetative stage. PCA was performed using cultivar-level mean DRI. Cultivar colors represent relative drought-response categories based on hierarchical clustering of D-values: drought-resistant response (red), moderately drought-resistant response (blue), moderately drought-sensitive response (green), and drought-sensitive response (purple)

PCA was performed using cultivar-level mean DRI values to visualize multivariate association patterns among cultivars under drought conditions. Principal component analysis (PCA) of drought resistance indices (DRI) derived from morpho-physiological and biochemical traits under drought stress revealed separation patterns among soybean cultivars according to their multivariate drought-response profiles (Fig. 6). The first principal component (PC1) explained 54.90% of the total variance and captured the major variation pattern among cultivars and DRI traits under drought conditions. The second principal component (PC2) accounted for 21.23% of the total variance and represented additional variation associated with differences in DRI trait patterns among cultivars. Together, PC1 and PC2 explained 76.13% of the total variance, indicating that the first two principal components captured a substantial proportion of the observed multivariate variation.

**Fig. 7:**
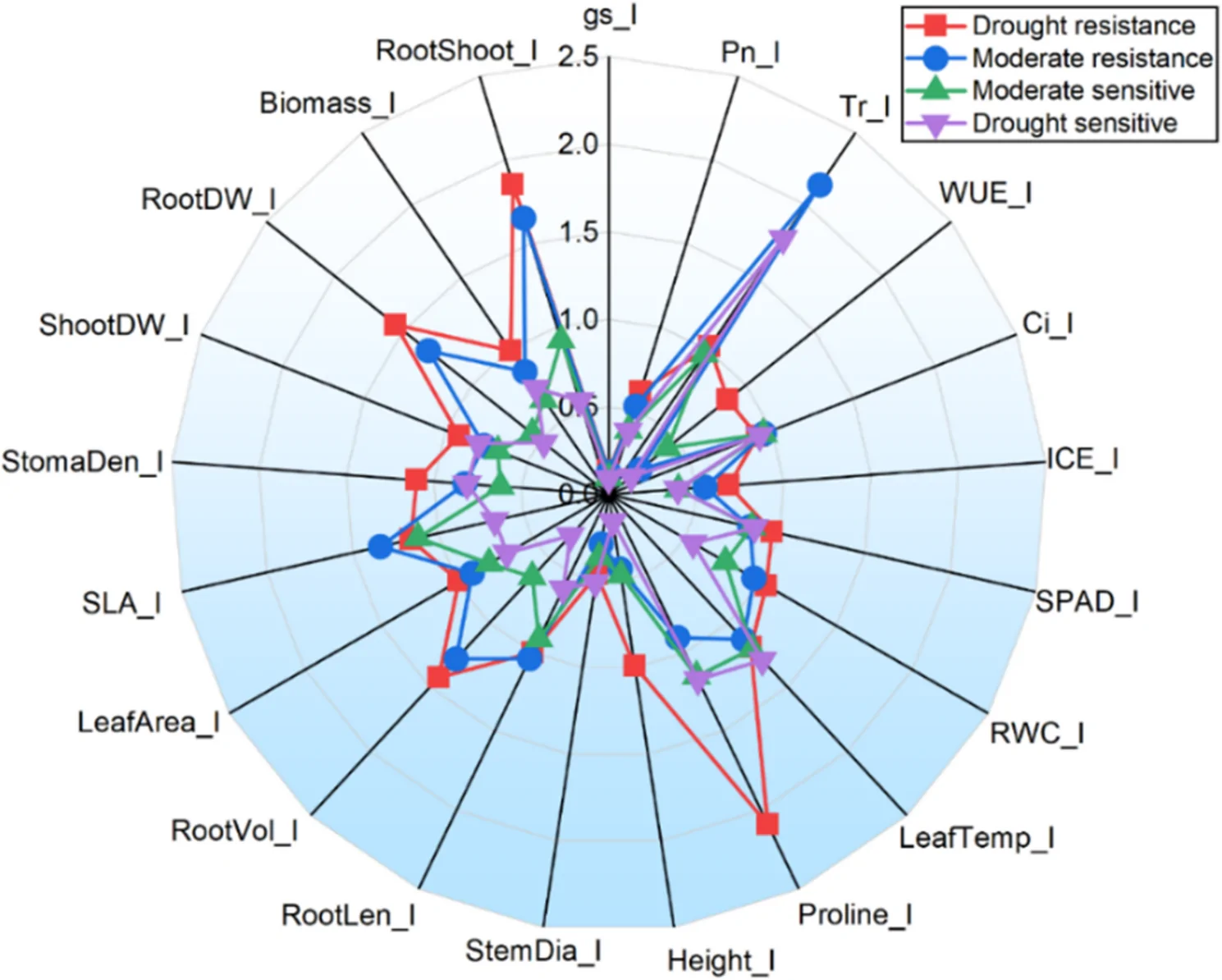
Radar plot illustrating differences in drought resistance index (DRI) trait performance among drought-resistance clusters under short-term drought conditions at the early vegetative stage of soybean

Several indices, including RWC_I, RootShoot_I, RootVol_I, RootLen_I, SLA_I, Height_I, Biomass_I, SPAD_I, Proline_I, StomaDen_I, and Pn_I, were oriented toward the positive direction of PC1, indicating stronger alignment with the major variation pattern represented by PC1. In contrast, LeafTemp_I, Tr_I, and Ci_I were oriented toward the negative side of PC1. This pattern was generally consistent with the DRI trait profiles shown in Fig. 7. Cultivars grouped within the drought-resistant cluster generally exhibited higher values of several root-related, physiological, and biochemical DRI traits, including RootVol_I, RootDW_I, RootShoot_I, Proline_I, RWC_I, StomaDen_I, WUE_I, ICE_I, and Pn_I. In contrast, the drought-sensitive cluster was more closely associated with higher LeafTemp_I and Tr_I values. The moderate resistance cluster showed DRI trait patterns generally comparable to the drought-resistant group, whereas the moderately sensitive cluster exhibited comparatively higher WUE_I and RootVol_I values together with relatively lower Tr_I values.

PC2 further differentiated cultivars and DRI traits according to their multivariate association patterns. The positive side of PC2 was associated with indices such as Pn_I, SPAD_I, StomaDen_I, ShootDW_I, and Biomass_I, whereas the negative side of PC2 was more closely aligned with WUE_I, RootLen_I, RootVol_I, RWC_I, and RootShoot_I.

The spatial distribution of cultivars in the PCA biplot was generally consistent with the hierarchical cluster analysis (HCA) based on D-values (Fig. 3b). Dering1 and Dega1 were positioned on the positive side of PC1 but differed along PC2, indicating differences in their multivariate DRI trait profiles. Detap1 occupied an intermediate position on PC1 and tended toward the negative side of PC2. Devon1 and Anjasmoro were located near the center on the negative side of PC1 and showed weaker alignment with several positively oriented DRI traits. In contrast, Biosoy1 was positioned furthest on the negative side of PC1 and was aligned with traits located on the negative side of the PCA biplot, including Tr_I and LeafTemp_I. Overall, the PCA revealed distinct multivariate association patterns among cultivars and DRI traits under drought conditions, highlighting coordinated variation among several physiological, morphological, and root-related drought resistance indices.

## Association analysis between DRI traits and D-value using partial least squares regression (PLS-R)

**Fig. 8:**
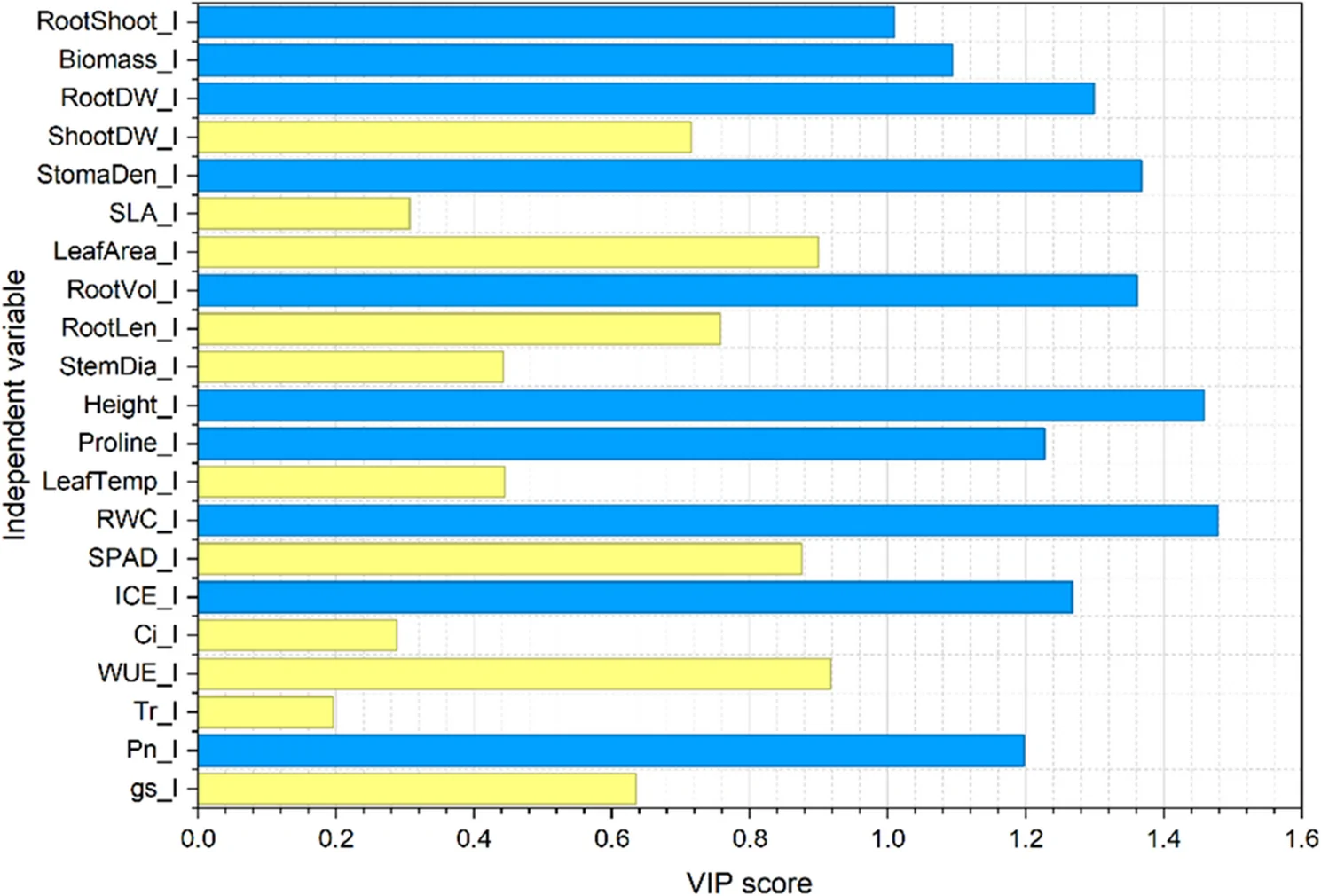
Variable Importance in Projection (VIP) scores of drought resistance index (DRI) traits in relation to the D-value based on partial least squares regression (PLS-R). Blue bars indicate traits with VIP > 1, representing relatively stronger associations with D-value variation within the analytical framework

Partial least squares regression (PLS-R) analysis was performed using drought resistance index (DRI) values from individual biological replicates to evaluate multivariate association patterns between DRI traits and the composite D-value under drought conditions (Fig. 8). A two-component PLS-R model was selected based on leave-one-out cross-validation (LOOCV) performance and model parsimony considerations. Additional latent variables produced only marginal improvements in Q² and RMSECV values; therefore, a two-component model was retained to minimize model complexity and reduce the risk of overfitting. The model produced calibration and cross-validation statistics of R² = 0.989 and Q² = 0.966, respectively, with RMSEC and RMSECV values of 0.013 and 0.023. The first two latent variables explained 43.6% of the total variation in predictor variables, with LV1 and LV2 accounting for 29.9% and 13.7%, respectively.

Variable Importance in Projection (VIP) analysis identified several DRI traits showing relatively stronger associations with D-value variation within the analytical framework. Traits with VIP values greater than 1 included RWC_I (1.49), Height_I (1.47), RootVol_I (1.37), StomaDen_I (1.36), RootDW_I (1.31), ICE_I (1.26), Proline_I (1.25), Pn_I (1.19), Biomass_I (1.09), and RootShoot_I (1.02). In contrast, LeafTemp_I, Tr_I, and Ci_I exhibited comparatively lower VIP values. The overall VIP patterns were generally consistent with the positive orientation of several root-related, physiological, and biochemical DRI traits along PC1 in the PCA analysis.

Overall, the PLS-R analysis highlighted coordinated multivariate association patterns among several physiological, morphological, and root-related drought resistance indices in relation to D-value variation under drought conditions. Given the relatively limited sample size and the composite nature of the D-value, these results were interpreted as exploratory association patterns within the analytical framework rather than as evidence of predictive or causal relationships.

## Discussion

The variability observed among traits (Table 1, and 2) and the variation in cultivar responses under drought conditions (Figs. 1 and 2) provided a basis for integrative analyses exploring multivariate association patterns among morpho-physiological traits [46] during early vegetative responses to short-term water deficit. Because the experiment was conducted under greenhouse conditions at the V3 stage using a five-day drought treatment, the present findings should be interpreted within the context of short-term drought-response dynamics rather than as evidence of stable drought tolerance across developmental stages or environments. The following discussion focuses on the application of the composite drought resistance index (D-value) to characterize variation in drought-response profiles among cultivars, examine association patterns among drought resistance indices, and evaluate coordinated multivariate relationships among physiological, morphological, and root-related traits under short-term drought conditions.

### Multivariate assessment of early-stage drought-response patterns using the DRI–D-value framework

The Drought Resistance Index (DRI) approach summarized through the composite D-value provides a multivariate framework for characterizing drought-response patterns [36, 45, 47] among soybean cultivars under the experimental conditions evaluated in this study. Differences in D-value profiles among cultivars reflected combined variation across multiple morpho-physiological and biochemical response traits that exhibited significant drought-related responses (Table 1, and 2). Variation in D-values among cultivars indicated differences in relative drought-response performance during early vegetative growth under short-term water-deficit conditions, with higher D-values generally associated with more favorable integrated response profiles under drought stress (Fig. 3a). Based on these values, hierarchical cluster analysis classified cultivars into four relative drought-response categories under short-term drought conditions: drought-resistant, moderately drought-resistant, moderately drought-sensitive, and drought-sensitive response groups (Fig. 3b).

The clustering results showed that cultivars with different D-value rankings could occupy either similar or distinct response categories. For example, the tolerant cluster included the two highest-ranked cultivars (Dering1 and Dega1), whereas the moderately tolerant cluster contained only the third-ranked cultivar (Detap1). The moderately sensitive cluster consisted of Anjasmoro and Devon1, while the sensitive cluster included Biosoy1. Although D-value ranking represents a continuous numerical scale, hierarchical clustering facilitated visualization of relative grouping patterns and multivariate similarity among cultivars within the composite index framework.

The placement of cultivars within each cluster was associated with combined variation among multiple morpho-physiological and biochemical traits integrated into the D-value framework rather than with any single individual trait. Previous studies have suggested that drought-response patterns are often associated with coordinated variation among plant water status, photosynthetic traits, root system characteristics, and metabolic responses under drought conditions [48–50]. In the present study, the DRI–D-value framework was therefore interpreted as an exploratory multivariate approach for examining coordinated drought-response patterns among cultivars during early vegetative exposure to short-term drought stress, rather than as evidence of definitive drought-adaptive mechanisms or predictive breeding performance.

### Coordinated association patterns among morpho-physiological traits under drought stress

Correlation analysis revealed that the inter-trait association structure differed between well-watered and drought conditions (Fig. 4). Under well-watered conditions, the D-value was primarily associated with general physiological traits, including Ci, Pn, RWC, proline, and StomaDen, which may reflect baseline physiological and photosynthetic status under non-stress conditions. Under drought stress, the association pattern shifted toward traits commonly associated with drought-response processes, including WUE, RWC, Pn, ICE, proline, and root-related traits (RootVol, RootDW, and RootShoot), whereas LeafTemp showed a negative association with the D-value. These shifts in association patterns suggest that early vegetative responses to short-term drought under the present experimental conditions were associated with coordinated variation among physiological, biochemical, and root-related traits involved in plant water status and gas-exchange responses [51, 52].

The stronger associations of root-related traits with the D-value under drought conditions further indicate increased coordination between aboveground and belowground response traits during water deficit. Greater root development and higher root-to-shoot ratios have frequently been associated with improved water acquisition and maintenance of hydraulic function under drought conditions [53, 54]. In the present study, positive associations among WUE, Pn, and ICE suggest that cultivars exhibiting relatively higher D-values also maintained comparatively favorable photosynthetic responses under drought conditions. In contrast, the negative association between LeafTemp and D-value may reflect relationships between leaf temperature dynamics and transpiration-related responses during drought stress [55].

The correlation network among DRI traits identified RWC_I as a central trait associated with multiple physiological and root-related indices. Its positive associations with gs_I, Pn_I, ICE_I, RootVol_I, RootDW_I, and RootShoot_I (Fig. 5) suggest coordinated relationships between plant water status, stomatal responses, photosynthetic traits, and root development under drought conditions [56]. Proline_I also showed positive associations with RWC_I and gs_I. These relationships may indicate that proline accumulation was associated with drought-response processes related to osmotic adjustment and maintenance of tissue water status. Previous studies have similarly reported associations between proline accumulation, cellular water balance, and stomatal regulation under drought stress [57–59]. However, because the present study evaluated responses at a single time point and did not assess proline synthesis or degradation dynamics, these interpretations should be considered exploratory rather than definitive evidence of adaptive physiological mechanisms.

Positive associations among Pn_I, ICE_I, and Biomass_I further suggest coordination between photosynthetic performance and vegetative growth responses under drought conditions [50]. Conversely, the negative associations of WUE_I with Ci_I and Tr_I may reflect stomatal and diffusional adjustments associated with water-conservation responses, consistent with previous reports describing stomatal and mesophyll CO₂ diffusion as major factors associated with WUE variation under drought stress [60]. On the root side, the positive associations of RootVol_I and RootDW_I with RWC_I, together with their negative associations with LeafTemp_I (Fig. 5), suggest relationships between root-related traits, plant water status, and leaf temperature responses under drought conditions. Similar associations between root development, plant water relations, and drought responses have also been reported previously [61].

Several DRI traits that showed strong positive associations with the D-value were also positively associated with one another (Fig. 5), indicating coordinated multivariate association patterns within the composite DRI–D-value framework [45]. Overall, the present findings suggest that early vegetative responses to short-term drought in soybean were associated with coordinated variation among osmotic, physiological, and root-related traits rather than with isolated responses of individual traits.

### Multivariate association structure of drought-response indices under short-term drought stress

Drought responses at the early vegetative stage involve coordinated variation among multiple physiological, morphological, and biochemical traits and therefore are not adequately represented by a single parameter [62–64]. In this context, principal component analysis (PCA) was used to explore multivariate association patterns among drought resistance indices (DRI) under short-term drought conditions (Fig. 6). By summarizing multiple DRI traits into principal components representing major patterns of variation under water deficit, PCA facilitated visualization of cultivar distribution within a multivariate drought-response space. Similar multivariate approaches have been widely used to characterize complex plant responses to drought and other environmental stresses [62, 63, 65].

The separation of cultivars along the principal components (Fig. 6) was generally consistent with the D-value classification (Fig. 3a), suggesting that differences in cultivar grouping patterns were associated with coordinated variation among multiple DRI traits. PC1, which explained 54.90% of the total variance, represented the major axis of multivariate variation under short-term drought conditions. Positive orientation along PC1 was associated with Dering1 and Dega1 together with several physiological, root-related, and growth-associated DRI traits, including RWC_I, RootVol_I, RootShoot_I, Proline_I, RootDW_I, Height_I, Biomass_I, SPAD_I, StomaDen_I, and Pn_I. These associations suggest coordinated relationships among plant water status, photosynthetic traits, root development, and vegetative growth responses under drought conditions [64–66]. In particular, the alignment of RWC_I and root-related traits along PC1 may reflect associations between root development and maintenance of plant water status during drought exposure [66]. Likewise, the positive orientation of Pn_I, SPAD_I, and StomaDen_I may indicate coordination between photosynthetic function and stomatal-related responses under water deficit [64].

In contrast, LeafTemp_I, Tr_I, and Ci_I were positioned toward the negative side of PC1 and were aligned with Anjasmoro, Devon1, and Biosoy1, which were classified as moderately sensitive to sensitive according to the D-value framework (Fig. 3b). Increased transpiration responses observed in several cultivars despite marked reductions in gs may have been associated with elevated leaf temperature and higher leaf-to-air vapor pressure gradients under drought conditions. Supporting this interpretation, cultivars showing increased Tr under drought also exhibited relatively higher VPD leaf and greater differences between leaf and air temperature (ΔTleaf–Tair), suggesting possible associations between transpiration responses, elevated leaf-to-air vapor pressure gradients, and leaf temperature dynamics under the present measurement conditions. Similar relationships between leaf temperature dynamics, transpiration behavior, and drought stress responses have been reported previously [55].

PC2, which explained 21.23% of the total variance, captured additional variation in multivariate response patterns among cultivars. The positive side of PC2 was more closely associated with photosynthetic and biomass-related indices, whereas the negative side was aligned more strongly with WUE_I and several root-related traits, including RootLen_I, RootVol_I, and RootShoot_I. Although Dering1 and Dega1 were both positioned on the positive side of PC1, their separation along PC2 suggests differences in the relative alignment of DRI traits within the same tolerance category. Dega1 was more closely associated with photosynthetic and biomass-related indices, whereas Dering1 showed stronger alignment with WUE_I and root-related traits, including RootVol_I and RootShoot_I. These patterns may indicate variations in multivariate drought-response profiles among cultivars that exhibited similarly high D-values under the current experimental conditions. Comparable variation in multivariate drought-response patterns has been reported previously in soybean; for instance, Mexican cultivars showed differential responses involving shoot biomass maintenance, enhanced root architecture, and modified root–shoot allocation [66].

Partial least squares regression (PLS-R) complemented the PCA analysis by evaluating multivariate association patterns between DRI traits and the composite D-value under early vegetative responses to short-term water deficit. A parsimonious two-component model was retained based on leave-one-out cross-validation (LOOCV), because additional latent variables produced only marginal improvements in Q² and RMSECV values. The observed R² and Q² statistics indicated consistency between calibration and cross-validation performance [67] within the present analytical framework. Traits with VIP values greater than 1, including RWC_I, Height_I, RootVol_I, StomaDen_I, RootDW_I, ICE_I, Proline_I, Pn_I, Biomass_I, and RootShoot_I, showed relatively stronger associations with D-value variation. Overall VIP patterns were generally consistent with the positive orientation of several physiological, root-related, and growth-associated traits along PC1 in the PCA analysis, indicating convergence between the two multivariate approaches [68, 69]. Comparable association patterns between physiological performance, root-related traits, and drought-response profiles have also been reported in sorghum and citrus under drought conditions [70–72].

Because the D-value was mathematically derived from integrated DRI traits, the PCA and PLS-R analyses were interpreted as exploratory evaluations of coordinated multivariate association patterns within the composite analytical framework rather than as evidence of independent causal mechanisms or predictive determinants of drought tolerance. Nevertheless, the consistency of association patterns observed across PCA, correlation analysis, and PLS-R suggests that early vegetative responses to short-term drought in soybean were associated with coordinated variation among plant water status, photosynthetic traits, and root-related responses under the experimental conditions evaluated in this study.

### Study limitations and future perspectives

The present study provides an integrative assessment of morpho-physiological drought-response patterns in soybean during early vegetative growth under short-term water-deficit conditions. However, several considerations should be acknowledged when interpreting the findings. The experiment was conducted under greenhouse conditions using a five-day drought treatment at the V3 stage, and physiological measurements were obtained at a single sampling point. Consequently, the observed responses primarily reflect early-stage drought-response dynamics under controlled conditions and may not fully represent drought responses across developmental stages, stress durations, or fluctuating field environments.

In addition, because the D-value represented a composite framework derived from multiple drought resistance indices (DRI), the correlation, PCA, and PLS-R analyses were interpreted as exploratory multivariate approaches for examining coordinated association patterns among drought-response traits rather than as evidence of independent causal mechanisms or predictive determinants of drought tolerance. Although consistent association patterns were observed among physiological, root-related, and growth-associated traits, several physiological interpretations, including those related to proline accumulation, transpiration behavior, and stomatal responses, remain inferential because temporal metabolic dynamics, hydraulic regulation, and recovery responses were not directly evaluated.

Furthermore, the present study did not assess reproductive-stage performance, yield stability, or multi-environment field responses; therefore, the observed cultivar differences should be considered preliminary indicators of drought-response potential within the experimental conditions evaluated. Future studies integrating temporal physiological measurements, hydraulic and metabolic analyses, reproductive-stage evaluation, and multi-environment field validation would help clarify the biological significance, stability, and agronomic relevance of the drought-response patterns identified in this work.

## Conclusions

Early vegetative drought-response patterns in soybean under short-term water-deficit conditions were characterized using an integrative Drought Resistance Index (DRI) and composite D-value framework. Among the evaluated cultivars, Dering1 and Dega1 consistently exhibited comparatively higher D-values and were grouped within the drought-resistant response category, whereas Biosoy1 showed the lowest D-value and was classified within the drought-sensitive category under the experimental conditions evaluated in this study.

The results further suggest that early vegetative responses to drought were associated with coordinated variation among plant water status, photosynthetic performance, stomatal traits, and root-related characteristics rather than with isolated responses of individual traits. Multivariate analyses indicated that cultivars belonging to similar D-value categories could still exhibit distinct multivariate response profiles. In particular, Dering1 showed closer alignment with root-related and water-status-associated traits, whereas Dega1 was more closely associated with photosynthetic and biomass-related indices, indicating variation in coordinated drought-response patterns among cultivars under short-term drought stress.

The PCA and PLS-R analyses showed generally consistent multivariate association patterns among physiological, morphological, and biochemical drought resistance indices. Traits including RWC_I, RootVol_I, RootDW_I, RootShoot_I, Pn_I, ICE_I, StomaDen_I, and Biomass_I exhibited relatively stronger associations with D-value variation within the analytical framework applied in this study. Collectively, these findings support the interpretation that early vegetative drought responses in soybean involve integrated multivariate coordination among water-status, photosynthetic, and root-related traits under controlled drought conditions.

Although the present findings remain specific to short-term greenhouse drought conditions at the V3 stage, the integrative framework combining DRI, PCA, and PLS-R may provide a useful exploratory approach for evaluating multivariate drought-response patterns during early-stage phenotypic screening. In this context, Dering1 and Dega1 may serve as promising candidates for further validation across developmental stages, environments, and agronomic performance conditions.

## Supplementary Information

Supplementary Material 1.

## Data Availability

All data generated or analyzed during this study are included in this article.

## Abbreviations

ANOVA: Analysis of variance
Ci: Intercellular CO₂ concentration
CK: Well-watered control condition value
CV: Coefficient of variation
D: Composite drought-response index (D-value)
DAS: Days after stress
DRC: Drought resistance coefficient
DRI: Drought resistance index
DW: Dry weight
E: Transpiration rate
FC: Field capacity
FW: Fresh weight
gs: Stomatal conductance
HCA: Hierarchical clustering analysis
ICE: Instantaneous carboxylation efficiency
LOOCV: Leave-one-out cross-validation
LV: Latent variable
PCA: Principal component analysis
PLS-R: Partial least squares regression
Pn: Net photosynthetic rate
PPFD: Photosynthetic photon flux density
Q²: Cross-validation predictive ability statistic
RCBD: Randomized complete block design
RMSEC: Root mean square error of calibration
RMSECV: Root mean square error of cross-validation
R²: Coefficient of determination
RootDW: Root dry weight
RootLen: Root length
RootShoot: Root-to-shoot ratio
RootVol: Root volume
RWC: Relative water content
SD: Standard deviation
SLA: Specific leaf area
SPAD: Leaf greenness index
StomaDen: Stomatal density
ShootDW: Shoot dry weight
Tair: Air temperature
Tleaf: Leaf temperature
Tr: Transpiration rate
TW: Turgid weight
VIP: Variable Importance in Projection
VPDleaf: Leaf vapor pressure deficit
WUE: Water-use efficiency
Xmax: Maximum value of a trait index
Xmin: Minimum value of a trait index
ΔTleaf–Tair: Difference between leaf and air temperature
