# Supplementary material for: Multivariate association patterns of morpho-physiological responses in soybean under short-term drought at the early vegetative stage using a drought resistance index framework
Source: BMC Plant Biol. 2026 Jun 13;26:1418. doi: 10.1186/s12870-026-09189-z (PMC13488001; doi:10.1186/s12870-026-09189-z)
Supplement: Supplementary file 1 [file 12870_2026_9189_MOESM1_ESM.docx]

**Supplementary Information**

**Fig. S1** Leaf vapor pressure deficit (VPD_leaf) measured using the LI-6800 portable photosynthesis system in six soybean cultivars under well-watered and short-term drought conditions at the early vegetative stage. Values represent mean ± SE of three biological replicates. Increased VPD_leaf under drought conditions may contribute to elevated transpiration responses in some cultivars despite reduced stomatal conductance

**Fig. S2** Difference between leaf temperature and air temperature (ΔT_leaf–air) measured using the LI-6800 portable photosynthesis system in six soybean cultivars under well-watered and short-term drought conditions at the early vegetative stage. Values represent mean ± SE of three biological replicates. Variations in ΔT_leaf–air under drought conditions provide additional context for interpreting transpiration responses among cultivars.

**Table S1**. Cross-validation performance metrics for PLS-R models with increasing latent variables

| Latent Variables (LV) | R²_cal | Q² (LOOCV) | RMSEC | RMSECV |
| --- | --- | --- | --- | --- |
| LV1 | 0.959 | 0.933 | 0.202 | 0.259 |
| LV2 | 0.989 | 0.966 | 0.113 | 0.197 |
| LV3 | 0.996 | 0.957 | 0.067 | 0.207 |
| LV4 | 0.998 | 0.969 | 0.040 | 0.177 |
| LV5 | 0.999 | 0.974 | 0.029 | 0.163 |
| LV6 | 0.999 | 0.976 | 0.026 | 0.153 |
| LV7 | 0.999 | 0.977 | 0.022 | 0.153 |

Calibration and leave-one-out cross-validation (LOOCV) statistics of partial least squares regression (PLS-R) models with increasing numbers of latent variables (LV), a two-component model was selected based on model parsimony and the marginal improvement in Q² and RMSECV values with additional latent variables.
